# Oral health and individuals with a lived experience of an eating disorder: a qualitative study

**DOI:** 10.1186/s40337-023-00841-9

**Published:** 2023-07-17

**Authors:** Tiffany Patterson-Norrie, Lucie Ramjan, Mariana S. Sousa, Ajesh George

**Affiliations:** 1 Australian Centre for Integration of Oral Health (ACIOH), School of Nursing and Midwifery, Western Sydney University, Penrith, NSW 2751 Australia; 2 Ingham Institute for Applied Medical Research, Liverpool, NSW 2170 Australia; 3 School of Nursing and Midwifery, Western Sydney University, Locked Bag 1797, Penrith, NSW 2751 Australia; 4 Translational Health Research Institute, Campbelltown, NSW 2560 Australia; 5 IMPACCT—Improving Palliative, Aged and Chronic Care Through Clinical Research and Translation, Faculty of Health, University of Technology Sydney, Broadway, NSW 2007 Australia; 6 School of Nursing, Faculty of Science, Medicine and Health, University of Wollongong, Wollongong, NSW 2522 Australia; 7 South Western Sydney Local Health District, Locked Bag 7103, Liverpool BC, NSW 1871 Australia; 8 School of Dentistry, Faculty of Medicine and Health, The University of Sydney, Sydney, Australia

**Keywords:** Eating disorder, Lived experience, Oral health, Non-dental health professional, Dietitians

## Abstract

**Background:**

Limited evidence exists describing the impact to oral health when living with an eating disorder and the availability of information or access to oral health services. This study investigated the perceptions of individuals with a lived experience of an eating disorder specifically to understand their needs and recommendations for improving access to early intervention and oral health promotion.

**Methods:**

Using purposive sampling a total of 12 semi-structured interviews were conducted with participants across Australia who had a lived experience of an eating disorder. A hybrid inductive and deductive approach to thematic analysis was used to construct salient themes and subthemes.

**Results:**

Most participants had experienced some oral health manifestation as part of their eating disorder hence, many felt quite knowledgeable on the topic. Following their eating disorder many participants felt confident in engaging with dental services, although, barriers including embarrassment, shame, and cost compromised access at times. Participants felt strongly that greater emphasis on oral health promotion during an eating disorder was important and this may be achieved by increasing the availability of resources and using trusted non-dental health professionals like dietitians.

**Conclusions:**

The need for oral health promotion while experiencing an eating disorder was evident, however, dentists can often be a costly option. Non-dental health professionals like dietitians working with clients with an eating disorder may be an acceptable alternative for closing this gap.

**Supplementary Information:**

The online version contains supplementary material available at 10.1186/s40337-023-00841-9.

## Abstract (plain language summary)

The link between eating disorders of any sub-type and poor oral health such as dental caries and enamel wear has been well established in the literature, but barriers such as embarrassment and dental fear can often deter individuals from seeking dental care. Non-dental health professionals such as general practitioners, nurses and allied health professionals including dietitians who work closely with individuals with an eating disorder may be able to play an active role in promoting oral health. The findings of this study provided insight into the experiences of individuals with an eating disorder and the impact this had on their oral health. Barriers to accessing oral health care included shame and embarrassment as well as the cost of dental services. However, many participants felt that having a trusted non-dental health professional such as their dietitian to provide education and referral to dental services would have been beneficial. Further research is needed to understand the requirements for dietitians and other non-dental health professionals to provide oral health promotion in eating disorder clinical areas.

**Supplementary Information:**

The online version contains supplementary material available at 10.1186/s40337-023-00841-9.

## Background

Living with a mental illness contributes a significant burden to the individual and members of society [1]. Globally, mental illness has been linked to premature death occurring nearly two decades earlier due to preventable physical conditions [2, 3]. It is estimated that nearly 1 in 8 individuals or 970 million people are living with a mental health condition with anxiety and depressive disorders considered the most prevalent [3]. Often co-occurring with other mental health conditions such as anxiety and depression, eating disorders (EDs) have been increasing in prevalence (3.5–7.8%) and incidence over the last few decades [3, 4]. Although EDs can affect anyone at any age [5], the adolescent period is the peak onset for EDs, affecting almost a third of adolescents in Australia every year [6–8]. Further, EDs has a likelihood of occuring more than once in an individual's lifetime [6].

The implications of an eating disorder (ED) regardless of sub-type are multi-systemic [9]. The impacts on the person can range from immediate acute health concerns including electrolyte imbalances, blood glucose irregularities, and gastrointestinal disturbance to long-term issues because of the chronicity of the condition, including impacts to reproductive health, neurological health and physical health [10]. There is also a significant impact on oral health that is overlooked and needs to be addressed. Two systematic reviews and meta-analyses exploring the relationship between EDs and oral health indicated an increased risk of tooth erosion (OR = 12.4, 95% CI = 4.1–37.5) and higher rates of decayed, missing, or filled teeth surfaces than the controls [11, 12]. Further reviews have also highlighted oral health complications including cheilitis, xerostomia (dry mouth) and enlargement of the parotid (salivary) glands because of the physical trauma and malnutrition associated with EDs [13].

Despite the risk to oral health, it is known that individuals with an ED or mental health illness are less likely to engage in dental services due to barriers such as shame, embarrassment, and fear [14–18]. Additionally, issues such as cost which effect the broader population in general, are cited as a significant barrier to accessing preventative and curative dental services [17, 18]. In Australia, dental services are available via the public health system. For adults, free dental care is accessible by those who are eligible for a health care concession card [19]. Children are also able to access free dental care in many states without the requirment of health care concession card and under the child dental benefits scheme (capped limit) [19, 20]. However, as with most public health dental services in developed nations, the system is often plagued by lengthy waitlists. Dental services are also accessible through private health care which is often financially inaccessible to the most vulnerable members of society [21].

To address this gap between dental services and vulnerable members of the population such as those with a mental illness, the role of the non-dental health professional has been explored as a means for providing pre-emptive oral health promotion (OHP). Non-dental health professionals such as general practitioners (GPs), nurses, or midwives servicing other at-risk population groups, including those with chronic disease or pregnant women, will often have frequent contact with their client group which makes them ideally placed for providing pre-emptive OHP [22–24].

The role of the dietitian has also been explored as a non-dental health professional who could play a part in pre-emptive OHP, especially in vulnerable populations such as ED. In a recent review of dietetic practice, dietitians were shown to already be incorporating OHP into at-risk population groups such as women, infants and children, the elderly, and individuals with Human Immunodeficiency Virus (HIV) [25]. While there may be a role for non-dental health professionals, there has been limited research afforded to explore the experiences of people with EDs regarding oral health and the need for pre-emptive oral health care. Furthermore, limited studies to date have explored the perceptions of people with EDs towards non-dental professionals, such as dietitians, playing a role in OHP.

## Methods

### Aim

This study aimed to explore the perceptions of individuals, with a lived experience of an ED, towards oral health. Specifically, the objectives were to understand the impact of oral health on EDs; their experience of care and receiving OHP; barriers and facilitators to accessing OHP or dental care and suggestions for improving access and knowledge around OHP for individuals with an ED.

### Design

This study was part of a larger sequential multi-phase mixed methods study that focused on developing an oral health model of care for dietitians working with individuals with EDs using the ‘adapted Medical Research Council (MRC) development phase framework’. Other phases of the larger study included a scoping review [25], a national survey of dietetic practice looking at OHP in Australia and a qualitative study with dietitians working in ED clinical settings exploring their knowledge and practices in OHP [26].

A descriptive qualitative approach was employed for this study as it allowed for detailed insight into those with a lived experience of an ED and their experiences and perceptions of oral health and access to OHP. Additionally, this qualitative study provided depth to the existing quantitative literature that examined the extent of oral health issues and access to dental care in populations with an ED [17].

### Recruitment

Purposive sampling via an opt-in approach was used to recruit participants. Individuals with a lived experience of an ED were contacted via the ‘Butterfly Collective’. The Butterfly Collective is an online lived experience network involving people across Australia who have a lived experience of an ED or are a carer, family member, or friend of someone with a personal experience. A flyer was drafted for potential participants as well as an online form to register their interest. Following approval from the manager of the Butterfly Collective, participants who belong to the group were emailed by the manager with a copy of the flyer and a link to register their interest. The registration form contained a brief overview of the study and the inclusion and exclusion criteria. Individuals who registered their interest via the form, and who were deemed eligible to participate, were contacted and emailed a consent form to sign and return with their preferred availability for a semi-structured interview via video/teleconference.

### Inclusion and exclusion

To be eligible for inclusion in the semi-structured interviews, participants had to meet the following criteria:

- Formally diagnosed with an ED by a healthcare professional (had a lived experience of an ED)
- saw a dietitian for their ED within the last 5 years
- aged 18 years or older
- not currently receiving any acute treatment for their ED
- able to communicate fluently in English

All other individuals were not eligible to participate. Ethics approval for this study was obtained from Western Sydney University Human Research Ethics committee, approval number: H13316. For confidentiality, all participants were assigned a pseudonym prior to analysis of the data. To assist with confidentiality, individual interviews rather than a focus group were chosen to maintain anonymity.

### Data collection

Semi-structured interviews were chosen for data collection as they allowed for the interviewer to facilitate questioning and be led by the interviewee as appropriate. The preliminary data from previous study phases (interviews with dietitians working in an ED clinical setting) were reviewed during the development of the interview guide (see Additional file 1: Appendix A) and assisted in informing the line of inquiry.

Interview questions focused on understanding participants’ experiences of any barriers or facilitators to accessing dental care or receiving OHP advice and understanding the acceptability of clients receiving OHP from their dietitian.

Additional demographic information including age, gender, highest education qualification, geographical location, type of ED, years living with an ED, and who provided the ED diagnosis were recorded. Interviews were conducted by the lead investigator (TPN) and reviewed by an associate investigator experienced in conducting qualitative interviews (AG). All interviews lasted approximately 40 min and were completed via video conferencing (Zoom) at a convenient time for the participants. Audio from the interviews were recorded with consent. The audio recordings were automatically transcribed into text and then professionally cleaned by a transcription service (Landmark Transcription).

Data collection was continued until consensus on data sufficiency (richness of data) was agreed upon [27, 28]. This was determined by preliminary scoping of the transcripts and regular meetings with the research team to discuss data patterns.

### Data analysis

Transcription files were imported into qualitative analysis software N*vivo version 12 for analysis [29]. A hybrid inductive and deductive approach to thematic analysis was undertaken [30, 31]. An a priori analysis was guided by the semi-structured interview guide and supported the construction of the initial major themes. Transcripts were de-identified and read by each author who independently coded their transcript. A consensus meeting was held to discuss patterns in the data. An inductive approach then followed to allow the research team to be guided by the participants’ voice to identify and understand the challenges and facilitators of obtaining oral health care and participants’ experience of receiving OHP from a dietitian. Data in the form of quotes were then segemented, coded and categorized to revise the a priori codes and inform the development of the major themes and subthemes.

### Trustworthiness

Many strategies were employed to enhance the trustworthiness of the data collection and analysis process. For transparency, a reflexivity/positionality statement of each author can be found in Additional file 1: Appendix B. To ensure findings were credible, the lead investigator was the participants' primary contact person and interviewer. This allowed for lines of rapport and trust to be built between the participant and interviewer to enhance communication. Weekly debrief meetings were held with (AG) to reflect on the interviews, identify any new patterns in the data, and confirm inductive codes. The latter supported rigourous analytical processes (analytical sufficiency) [27, 28]. Due to time constraints and to reduce participant burden, member checking was not used in this study, however, transcripts were professionally cleaned and edited to ensure dependability of findings.

Additionally, all interview transcripts were reviewed by the lead investigator and a sample of transcripts were reviewed by the co-investigators AG, LR and MSS. A coding meeting was held to discuss final themes and data sufficiency. Finally, the Standards for Reporting Qualitative Research (SRQR) was used to guide reporting of this qualitative study [32].

## Results

### Characteristics of participants

A total of twelve individuals identifying as female with a lived experience of an ED participated in the semi-structured interviews (denoted as pseudonyms P1-P12 hereafter). Participants had a mean age of 36.9 years old and had mean of 15.5 years experience with an ED. Anorexia Nervosa or Bulimia Nervosa was the most commonly experienced ED (n = 8), followed by binge eating disorder (n = 2), Avoidant/restrictive food intake disorder (AFRID) (n = 1) and Other specified feeding and eating disorder (OSFED)(n = 1). The majority of the participants were employed on a part-time basis (n = 5) and others were either full-time (n = 3) or unemployed (n = 4). The highest qualification amongst the group of participants was a Masters (n = 1), followed by post-graduate degrees including a bachelor's degree and post-grad certificates. Participants were recruited from various states across Australia including New South Wales (n = 6), Victoria (n = 2), Queensland (n = 1), South Australia (n = 2), and Western Australia (n = 1).

Through the hybrid analysis, three major themes and ten sub themes were identified (Table 1).

**Table 1:** Major themes and subthemes

Major themes	Sub themes
Experiences and awareness of ED related oral health	Oral health issues encountered Impact on oral health and general health Facilitators to oral health Interactions with dentists
Barriers and challenges to oral health	Limited focus on oral health by professionals Embarrassment, shame and taboo Dental costs and access
Suggested model of care	Oral health promotion and resources Building capacity of dietitians Appropriate and affordable dental care

### Experiences and awareness of ED related oral health

#### Oral health issues encountered: ‘erosion…the front four teeth were the worst’

Many participants recalled numerous oral health concerns they experienced. Enamel wear or acid erosion was the most identified oral health issue. Many highlighted that this was largely due to vomiting practices, however, some also explained that ice chewing led to erosion: ‘*I got erosion, yeah erosion. Really the front four were the worst but eight* (teeth) *just started fading away getting smaller and yellower and yellower’* [P4].

In addition to erosion, participants also experienced bad breath, tooth damage and pain.

‘*When I was vomiting quite regularly, the acids from throwing up is quite damaging to teeth…but also the tools* (equipment used to assist with self-induced vomiting e.g. toothbrush) *that I’ve used in the past… tools I would use to throw up which can lead to chipping and things like that…’* [P7]

For the many who experienced oral health concerns, participants reported feeling that the extent of their symptoms were burdensome and overwhelming.

‘*it was just so hard to keep on top of everything* (symptoms associated with ED) *and it was just very overwhelming…it was just so exhausting to do anything because I don’t know, you were scared what’s going to happen next…’* [P2]

#### Impact on oral health and general health: ‘it can affect your overall health and quality of life’

Nearly all participants discussed awareness of the impact of ED on oral health: *‘it massively affected my oral health’* [P4], ‘…*I know that it completely mucks up people’s teeth, destroys them, I think’* [P12]*.* Participants described an understanding of the effects of limiting nutritional intake or compromised vitamin and mineral intake e.g. calcium on teeth health or the overconsumption of high-sugar food items and acid washing of teeth.

‘*The only thing that I can think of from knowledge is, I guess when you don’t eat or you eat certain types of food, and I think when you vomit as well, it damages your teeth because of the acid.’* [P6]

A few participants also drew attention to the impact on mental health because of poor oral health. Participants expressed changes in self-esteem, quality of life and anxiety:

‘*It's pretty detrimental. For example, I had a top tooth that was infected, and the pain shot into my sinuses… it can affect your overall health and quality of life especially if you lose a lot of teeth.’* [P1]

As a result of changes to their oral health some participants explained needing to change their dietary choices and oral hygiene practices: ‘*…this has only been something in the last 18 months that I’ve experienced this to the point where I’ve needed to change toothpaste. Prior to that, yeah I was definitely avoiding cold foods…’* [P8].

However, some noted that they were unaware of the impact that ED can have on oral health: ‘*I didn’t know what was going to happen to me…Could something orally happen?’* [P2]. Interestingly, one participant noted that ‘*I think that at the time maybe I wasn’t so worried about the condition of my teeth, but now it’s definitely something that I’m quite aware of’* [P8].

#### Facilitators to oral health: ‘I’m very committed to being physically well. My oral health would be part of that…’

Participants described a variety of factors that supported them in seeking oral health services. Some participants commented ‘*…I want to eat now, which is a great sort of recovery…it was annoying to not be able to eat ice cream or have grapes from the fridge so I was quite motivated to be able to do that* (eat) *without it being an unpleasant experience’* [P8] while for many other participants, oral health priority is: ‘…*a lot higher these days…’* [P2] and has become more of a priority to their health following the remission of their ED.

‘*If I was creating a list of my physical health, it would be at the same level as everything else. I’m very committed to being physically well. My oral health would be part of that…’* [P11]

A few participants also raised issues such as being self-conscious of one's teeth, the impact of vomiting and the importance of teeth and self-image and this being a motivating factor for protecting their oral health and seeking out dental services:

‘*Yeah, I think teeth are pretty important… I think these days there is a lot more emphasis on people having nice-looking teeth and not just damaged teeth… there is more of a hyper-focus awareness on beauty and it is probably more distressing for people now in the quite bad parts of their eating disorder. It’s more than it was at the time…’* [P7]

This participant also went on to note, that maintaining the condition of their teeth and oral health became so important to them that it was a major factor influencing their purging behaviours: ‘*…I’m visibly noticing the damage in my teeth and then being told it’ll get worse if you keep doing that, so it’s become incredibly important. In fact, it’s probably one of the major reasons that I don’t throw up anymore even if I do have the urgency because I don’t like the damage it could do to my teeth…’* [P7].

Interestingly, a few participants also reported that they were ‘frightened’ into caring for their oral health by their dentist and this was a motivating factor that encouraged them to pay more attention to their oral health and have regular dental checkups.

‘*I pretty much got frightened over a period of time to look after my teeth in that way because my dentist told me, it was probably a scare tactic but he said to me, ‘’if you don’t look after your teeth, they’ll drop out’’…’* [P12]

Generally, many participants felt confident in managing their personal oral health and visiting a dentist if they had an oral health concern. For some, this was because they had never previously experienced an oral health concern, whereas for others, it was because they were seeking preventative care to ensure they limited their risk for developing oral health issues.

‘*…I would say I’m fairly confident. I think I’ve just got naturally good teeth and I brush them everyday and I’ve never had any issues.’* [P10]

One participant reported she was scared initially by the financial burden associated with seeking help for oral health, however, with time feels she has become more confident: ‘*I think I am more confident now than I was at being able to deal with it, I’ve seen a lot of fears and reservations and a lot of financial insecurity.’* [P9].

#### Interactions with dentists: ‘I’ve started seeing a new dentist who is amazing’

For the most part, many participants reported a positive experience visiting their dentist. As reported by one participant: *‘… the most helpful person has been my dentist…’* [P4]. Many participants appreciated the explanation of what was happening to their oral health and the OHP provided. This encouraged them to continue seeking oral care:

‘*…my current dentist I think I had two or maybe even three consultations with me before even touching my teeth and was just about what I needed, what I wanted, what I could financially do and afford and let me make the most of my private health insurance’* [P4]

Additionally, other participants felt their dentist was empathetic and went above and beyond to assist with providing alternative options that were sensitive to their individual needs:

‘*I’ve started seeing a new dentist who is amazing and she told me so much that I have never known about before…explaining everything that she's doing and what’s caused it and how we can prevent it in the future. She’s actually going to set me up for a consultation with an oral health* [therapist]’ [P1]

### Barriers and challenges to oral health

#### Limited focus on oral health by professionals: ‘no one’s ever mentioned oral health’

Generally, participants reported that they received little to no information or resources from healthcare providers regarding their oral health ‘… *from my experience of seeing eating disorder practitioners, you tend to get very little written information… I’ve had an eating disorder for a long time, but it’s very rare I’m given information*’ [P10].. One participant commented that they felt the GP did not have adequate knowledge to discuss EDs: ‘*They’re basically just googling it while you’re sitting in their office and that’s actually happened to me a few times… that’s quite frustrating…your GP is meant to be your central person in the care team’* [P11].

Further, for those who received support primarily from a GP, most reported that GPs had limited time to discuss oral health.

‘*You know even the GP, ‘cause you have such a limited time with them…it really focuses on you heart health, your blood pressure, all that kind of stuff…’* [P9]

This sentiment was echoed by participants about the wider multidisciplinary team.

Participants described never having received any OHP resources or education: ‘*…I have a healthcare team, no one’s ever mentioned oral health or really the impacts of an eating disorder on your teeth. No one has ever said ‘’hey this can happen because of your eating disorder’’…’* [P11].

In terms of identifying appropriate healthcare professionals to discuss oral health concerns with, most participants felt ‘…*we didn’t really know anyone* (dental services) *we could go to…’* [P11].

#### Embarrassment, Shame and Taboo: ‘I’m embarrassed and ashamed that I’d done this to myself…’

Another area highlighted by many participants was embarrassment, anxiety and shame around disclosing ED-related impacts on oral health. For some participants, it began before setting foot into the dental clinic as their embarrassment would deter them from making an appointment or considering visiting the dentist: ‘*I’m embarrassed and ashamed that I’d done this to myself…’* [P2]. For others, they were hesitant to show the dentist their mouth due to judgement: *‘they can tell the grinding as soon as they open my mouth, they go, “oh my goodness”… I think that I’m a bit embarrassed to mention I’ve had any disorder’* [P6].

Others discussed avoidance of dental services, particularly during their ED for worry of judgement or their parents finding out about their ED.

‘*I didn’t want to go when I was a teenager because my parents would likely be in the room and they didn’t know, they didn’t know about the behaviours* (ED behaviours)*, so I didn’t want them to find out that way… I didn’t want them to judge me for having poor oral hygiene, even though I brushed my teeth regularly. A large part of it was just embarrassment…* ‘ [P9]

Some participants also raised the challenge of oral health being a ‘taboo’ topic or something that may not be appropriate for discussion in certain circumstances. One participant reflected on discussing the topic in a social situation and felt that oral health may be a taboo subject as it was never something that was discussed among friends:

‘*…It’s never really been a discussion with friends. I find discussing… health with friends is more bloating these days or iron levels… I can’t recall actually having a conversation with someone like, ‘‘I wanna see my dentist like this is what works for me’’…I think it’s something that’s maybe taboo ‘cause its teeth…’* (P2)

#### Dental costs and access: ‘the dentist is quite expensive’

There were many factors raised by participants for seeking dental care. Costs specifically, were a significant theme that participants discussed. Few participants acknowledged that if it wasn’t for their private health insurance or parents financially assisting them to seek dental treatment accessing oral care would be a challenge.

‘*I know that at* (healthcare fund) *where it’s no gap* (healthcare fund covers the entire cost of dental visit) *treatment is covered by my extra dental insurance, I’m looking after my teeth… it’s good’* [P12]

The burden of cost and being weary of visiting the dentist and finding out about requiring expensive dental treatment was another key barrier for many participants: ‘*I was too afraid to go for that entire time, partially because I knew the symptoms were still occurring and partly because I knew how expensive it would be.’* [P9].

‘*I’d probably go twice a year, but really struggle with it financially, because even preventative, the dentist is quite expensive. It can be over $250 just for preventative treatment. Yeah, I think money is a barrier’* [P12]

Access was another barrier discussed by participants. Although the lack of availability of dental services in rural locations was raised by one participant, two other participants raised other concerns such as injury and neuropathy as a consequence of their ED that has impacted their ability to drive and therefore, their ability to access dental services.

‘*…I can no longer drive as a consequence of my eating disorder. I developed three neuropathies. One of them is an optic neuropathy, so I can’t drive anymore.’* [P1]

Others chose to access free dental services via the public system however, a couple had certain perceptions on the waitlists to engage in an initial appointment or wait periods between appointments which were considered barriers.

‘*… there's a huge waitlist for people to access public dental services…I’d say you’d be very lucky to get in within a year…’* [P5]

Accessing dental services during the COVID-19 pandemic and subsequent lockdowns also affected the availability of dental services and restricted preventative therapies for a few participants. One participant mentioned: *‘ …I didn’t see the need to go see a dentist except when my tooth broke and that, I had to go in as an emergency…’* [P2]. Another recalled their experience of having their dental appointments cancelled: *‘ I think I had* (an) *appointment in the first lockdown that we had in Sydney. I had an appointment in the middle of that and that got cancelled because of COVID…’* [P12]. One participant also described their experience of managing their oral health during lockdown: ‘*I find it a bit of a drag to look after my teeth every day. On occasion, I let that slip during COVID when I was at home a lot more’* [P3].

Lastly, participants also discussed the impact of fear or avoidance of dentists. Some participants reported a dislike of visiting the dentist, fear of dental procedures and anxiety. In saying this, most were unable to identify a particular reason for feeling this way.

‘*I still don’t love seeing the dentist, even though he's great. I don’t know, something about it. No one likes the dentist. I don’t really know what it is but I don’t like it’* [P4]

### Suggestions for a model of care

#### Oral health promotion and resources: ‘if I was given a leaflet… It would have to be quite specific to the eating disorder I had’

Most participants were interested in learning more about the impacts of oral health and felt that discussing oral health would assist in encouraging dental visits. Importantly, most participants reported that oral health education was a valuable topic, especially if it would assist with early prevention of oral disease complications related to ED:

‘*I think that it needs to be discussed from the point of intake…*(it is) *a very dangerous thing* (poor oral health)*. The same that we talk about things like water loading or all those other kinds of behaviours that you may have a bit more chance of actually reducing from the very beginning.’* [P9]

Some participants felt that all health professionals should be talking about oral health, especially when working with ED.

‘*If it came up in conversation that it could be affecting my teeth or my oral health in general and someone suggested to me, a professional suggested to me, that it might be a good idea to go and get a dental check, I think that would be fine.’* [P12]

Furthermore, many participants described a desire for wanting ‘take home’ resources that would help increase their understanding of the impact of ED on oral health: *‘I think how each eating disorder can affect your oral health, the long term consequences of not fixing it and what you should do as the next step…’* [P1]. In particular, some participants reported they would prefer to have a specific resource for their ED in place of a generic resource or one targeted at one of the major EDs such as anorexia nervosa: *‘if I was given a leaflet about bulimia and oral health, I wouldn’t read it because I don’t have that particular eating disorder. It would have to be quite specific to the eating disorder I had.’* [P12].

In terms of information provided by the resource, many participants wanted to know: ‘*…what the risk factors are…’* [P10] and the possible management strategies: ‘…*probably just things to look out for. Yeah just any early warning signs of issues or things that I could be at increased risk of because of my disorder. I think that would be really helpful…’* [P11].

Building capacity of dietitians: ‘*…dietitians do tend to look at things about health holistically’.*

Many recalled having a positive experience with their dietitian citing compassion, the ability to provide nutrition advice, counselling and empathy as element of care they appreciated:

‘*…it’s like she gets how my brain works and is really positive, and if I tell her I’m scared, she tells me how to overcome that. It’s a really positive experience…’* [P1]

When posed with the question of how they would feel if a dietitian was to discuss OHP with them while experiencing an ED, the overwhelming majority of participants felt positive about this saying: ‘*…dietitians do tend to look at things about health holistically so why wouldn’t that include some knowledge about dental health…’* [P12] and ‘*…I can’t think of anyone else* (dietitian) *who really would have done that role* (OHP)’ [P9].

Many participants expressed that the dietitian could be a trusted professional to have this discussion with: ‘*…I would definitely trust that.’* [P2]. This sentiment was echoed by many others who also felt they could trust their dietitian and would trust the oral health information provided: ‘*…absolutely, especially my dietitian now, but even my dietitian then. I had a lot of trust in their knowledge, and the kind of information that they would give to me…’* [P4]. Few participants did mention that they would not be receptive to receiving OHP from their dietitian as: *‘…I don’t know, I think I would feel sort of criticized and like it’s none of your business kinda thing. I just think that would be my reaction…’* [P3].

Participants went on to provide recommendations for dietitians if they were to take a more active role in OHP. They expressed that dietitians’ should talk about oral health in relation to nutrition: ‘*not so much ‘‘do you have holes in your teeth?’’ things as well like, ‘‘is eating painful in your jaw?’’ and stuff like that ‘cause that affects food intake, ‘cause that’s what the dietitian to me is for…’* [P5] and that talking about oral health is a really important role that dietitians should play: ‘*I think that’s important and I think it should be coming from them because they are both mental and physical health’* [P4].

Some particpants also highlighted the importance of dietitians having access to training in oral health. These participants felt that ‘*…it* (oral health training) *would be a good part to have included in dietitian training’* [P12] however, *‘…nothing really in-depth, but just to have enough knowledge to know when it’s a good idea to bring it up in conversation…’* [P12]. One participant also highlighted the importance of reassuring confidentiality with clients: *‘…just acknowledging that they don’t have to share anything with them that they don’t want to… and also thanking that person for opening up…’* [P5].

#### Appropriate and affordable dental care: ‘nominated dentists who are quite knowledgeable with eating disorders’

The most salient recommendation by participants for dental clinical care was ensuring that they were connected with dentists who were specialized or experienced in providing care to individuals that had experienced an ED.

‘*…I’m sure they’re all great, but preferred ones that can be suggested that might be helpful rather than just picking out someone at random and then just hoping that they’ll be quite good with someone with an eating disorder teeth related issues.’* [P7]

A few participants also felt that it was important for the dentist they were referred to, to be non-judgemental and person-centered: ‘*I know it might sound a bit like why does the dentist need to be person centered but it made such a difference for me’* [P4].

Additionally, some participants also discussed the benefit they would receive if they were to have financial support for accessing dental services from medicare: ‘…*I feel as part of an eating disorder management plan, perhaps medicare should give some rebates on dental work potentially, and that would help with financial access.’* [P1].

## Discussion

The findings of this study contribute to a growing body of evidence which highlights that EDs can have a detrimental and irreversible impact on oral health. The results indicate that individuals with an ED may experience oral health concerns and this can have a significant impact on their self esteem and confidence. Barriers including embarrassment and cost of dental services may limit them engaging in treatment however, indivduals with an ED are open to the idea of pre-emptive OHP provided by non-dental health professionals like dietitians. This study is also the first dedicated qualitative study to investigate how oral health is addressed and managed by this population group and specifically, what individuals with a lived experience of an ED identify as important to managing and addressing their oral health concerns. Although some research does exist exploring the experiences of ED clients and receiving dental care [17], little evidence to date has gone beyond a survey design to explore in-depth the experiences of individuals with an ED and their oral health experience or how dental services may be best tailored to suit their needs.

Many participants were reliant on their experience with an ED as their main source of knowledge or awareness of the link between EDs and oral health. Although their knowledge was mostly ED subtype-specific, many were aware of the impact of malnutrition or trauma and the link to oral health. These findings confirm that of other quantitative studies where individuals with an ED have reported being aware of dental concerns linked to EDs such as tooth erosion (76%), risk of oral cancer (38%) and enlargement of salivary glands (30%), however, they were less knowledgeable on specific information such as where erosion was more likely to occur. In a larger cross-sectional survey of individuals with an ED by Dynsen et al., similar findings were reported, where majority of the participants (73%) were noted to have some knowledge on ED and oral health [17]. This finding is underpinned by participants linking their oral health status closely to their mental health and sense of self. Looking more broadly at mental health conditions, many reviews exploring the link between oral health and mental health have the same message, ‘there is no mental health without oral health’ which informs an important gap [33, 34]. If individuals with an ED continue to have limited knowledge and awareness to see and manage deteriorating oral health, they are likely to be at risk of this impacting their overall mental health.

When coupled with well-known dental fear or anxiety, embarrassment and financial constraints around accessing oral health, individuals may be faced with a significant number of barriers that may deter them from accessing dental services altogether [14, 35, 36]. Further, the increasing cost of dental services, lengthy public waitlists, and the stigma of mental health conditions must also be considered [37, 38]. As reported by Dynesen et al., only 15% of respondents engaged in dental services while experiencing their ED [17]. Additionally, participants in this study largely reported only attempting to discuss oral health with care providers or visiting a dentist after their ED. Therefore, given the evidence that exists which shows there is a significant risk to oral health during an ED, the non-specific awareness of individuals with an ED and the link between poor oral health and declining mental health, it is pertinent to review options that may improve access and knowledge around oral health.

Considering all of this, a promising finding was the significance participants placed on early intervention and prevention of oral health, to the point where some stated it became the impetus for reducing or stopping purging behaviours. This notion of protecting oral health was also noted in a survey of individuals with an ED, where nearly one-third of clients reported engaging in protective oral health behaviours such as rinsing with a neutralizing solution, avoiding brushing immediately after purging and reducing intake of acidic food [17]. This was also reported by individuals experiencing a mental health condition in a qualitative study by Ho et al., who also felt motivated to care for their oral health [35]. Furthermore, participants in this study also expressed a need to have oral health information provided to them and the choice to review options to seek dental care. Identifying oral health as a priority provides encouragement for health professionals to take more of a role in promoting oral health in practice. This is significant when survey results from other studies show participants stating that health professionals involved in the care of those with an ED should be promoting oral health and, have felt ‘cared for’ or their ‘needs recognized’ when they have received OHP advice [17]. This was also reiterated in our findings. However, further literature on facilitators to oral health in populations with a mental health condition is limited or only considers facilitators from the view of the health professional [38, 39]. In saying this, as per the experience of participants in this study, a survey of clients with an ED and a qualitative study of participants with broader mental health issues, many individuals agreed that when engaging with dental services either via dental or non-dental professionals they generally had a positive experience [17, 35, 38].

The acknowledgement of merit in knowing more about oral health by participants for early intervention or prevention is a finding that should motivate health professionals into engaging with these consumers. However, the issue of dental fear, or the stigma around mental health and oral health may always be an issue for some [35, 39]. An alternative to encouraging visits to dental clinics may be to consider non-dental health professionals as a vehicle for OHP as suggested in our findings. In this study, participants identified the dietitian as a trusted health professional and someone who had either discussed oral health or someone who could play a role in promoting oral health. There is evidence to suggest that the scope of practice of dietitians has been expanding across many clinical settings including dietitian-first gastroenterology clinics, where waitlist times have been reduced and screening processes improved with earlier symptom management. [40–42]. Similarly, in a review by Rieter and Graves, the authors comment on the role nutritionists can take in providing nutrition-related oral health advice and referral to dental professionals [43]. This is further reiterated in a scoping review and qualitative study of dietitians and their role in OHP with ED clients, where recommendations from dietetic bodies and dietitians themselves, express that OHP with this client group is a role they can, and importantly, should be championing [25]. Further, in other clinical areas such as aged care and women, infants and children, dietitians were already providing basic oral health screening, referral to dental professionals and education However, an important finding reiterated in a review of these studies was the lack of training and resources available [25].

In awareness of the importance of oral health in ED, a resource was developed by key ED stakeholders, academics and the New South Wales Health Department [44] for healthcare professionals to provide individuals with an ED information about risk factors, symptoms, prevention and where to seek support. Training and continued professional development (CPD) opportunities continue to be lacking for dietitians in this area [25, 26]. Using non-dental health professionals as a vehicle for OHP and referral to dental services is not a new concept. Other qualitative studies and reviews have explored the role of alternative health professionals such as nurses, midwives and general practitioners in vulnerable population groups such as chronic illness and pregnancy [22, 24, 45, 46]. The outcomes of these studies provide promising findings that using non-dental health professionals can be accessible and acceptable, but also a cost-effective option for consumers [47].

Another key finding raised by participants in this study, as well as in other surveys and reviews was the desire for specially trained dental professionals that had experience in working with clients with an ED or other mental health conditions [14, 35, 48]. Further, this desire for specialized training is also acknowledged in studies involving dentists [49]. In a Norwegian survey, 76% of dentists felt that they required specific ED training and 38% felt that dentists with specialized training in ED should be the ones to manage clients with an ED [50]. As acknowledged by Crowe et al., despite nutrition (or lack of) having a significant impact on oral health, the implication of impaired nutritional intake is rarely acknowledged in undergraduate curricula for dental students [51]. Further, a study of Canadian dentists found that they were largely not comfortable discussing sensitive health issues with their patient’s [52]. Hence, it should be considered that in addition to CPD training for existing dental professionals, attention should be given to providing education at the undergraduate level to improve the knowledge and awareness of mental health conditions such as ED in dentistry and other health graduates.

When coupled with well-known dental fear or anxiety, embarrassment and financial constraints around accessing oral health, individuals, especially those experiencing a mental illness [37, 39, 53], may be faced with a significant number of barriers that may deter them from accessing dental services altogether [10, 29, 30]. The increasing cost of dental services, lengthy public waitlists, and the stigma of mental health conditions must also be considered [31, 32]. Consideration of a non-dental health professional model for promoting oral health among individuals with an ED may allow for financially friendly access to oral health information and basic preventative screening. As shown in an economic evaluation of a midwifery-led OHP intervention [47], once professionals received training and were confident to implement this into practice, non-dental health professionals can provide an economically viable option for preliminary screening, education and referral [47]. The outcomes of these studies provide promising findings that using non-dental health professionals can be accessible and acceptable, but also a cost-effective option for consumers [19].

When reviewing the findings of this study it is important to consider the limitations. Although the effort was made to recruit a sample of participants from across Australia, with a range of years of experience with ED and varying demographics such as gender, choosing a qualitative methodology to obtain a deeper insight into the experiences of individuals with an ED the findings does impact generalisability of our findings to the broader population with a lived experience of an ED. Further, participants were recruited from a pool of individuals with a lived experience of an ED who had registered their consent to be part of a target group that could be contacted for recruitment to research studies. Hence, those who opted to participate may have had a vested interest or experience to share that may increase the risk of bias in the findings. It should also be noted that a few participants had family members who worked in the dental profession (i.e. Dental hygienist) which may have also contributed to their interest to participate in this study.

## Conclusion

EDs can have a significant and irreversible impact on oral health which research acknowledges is linked to self-image and mental health. Although individuals who have a lived experience of an ED are largely accepting and motivated to seek further information for early intervention and protection of their oral health, there is a significant gap in the oral health care they received from their ED care providers. To improve access to OHP for this vulnerable population group it is essential to consider upskilling key non-dental health professionals. Support for these professionals needs to consider pre-emptive education and resources for increasing awareness around oral health complications associated with ED and improving ED and trauma informed training for dentists. Further, informative guidelines to support both non-dental health professionals and dentists in providing holistic pre-emptive oral health care to individuals who present with an ED is required.

## Supplementary Information

**Additional file 1:** Semi-structured interview guide.

## Data Availability

All data generated or analysed during this study are included in this published article.
