# Supplementary material for: Oral health and individuals with a lived experience of an eating disorder: a qualitative study
Source: J Eat Disord. 2023 Jul 17;11:121. doi: 10.1186/s40337-023-00841-9 (PMC10353210; doi:10.1186/s40337-023-00841-9)
Supplement: Supplementary file 1 — Additional file 1: Semi-structured interview guide. [file 40337_2023_841_MOESM1_ESM.docx]

Supplementary file:

Appendix A: Semi structured interview guide: Individuals with an eating disorder (ED)

**Demographic questions**

- Eating disorder diagnosis:
- How long have you had an eating disorder for?
- Who diagnosed you with an eating disorder?
- Age:
- Gender:
- Highest attained qualification:
- Type of employment: e.g. Full time, part-time, casual, not employed
- State currently living in/ postcode if appropriate:

**Knowledge**

- Do you think eating disorders affect your general health, how?
- Do You think eating disorders affecting your oral health, how? Are there any risk factors?
- How do you think oral health affects your general health?
- (self esteem, confidence, anxiety, appearance)
- What are things you can do to protect your oral health? (not brushing immediately after vomiting, flossing)

**Attitudes**

- Do you have any oral health concerns?
- How important is talking about oral health with a health care professional when you have an eating disorder? Is it a priority? If not, what are other high priorities and why is not oral health less important in comparison to these?
- What experience have you had with health professionals regarding your oral health?
- Has anyone ever talked to you about your oral health?
- Have you ever disclosed oral health issues to anyone ?
- What information has your dietitian given you regarding your oral health?
- What are your feelings about your dietitian talking to you or asking about your oral health
- What can dietitians do to help you with your oral health?
- Do you have any oral health fears or anxieties? How can this be managed?
- How do you feel about dietitians providing you information on oral health promotion/prevention?
- What are your thoughts on dietitians asking some screening questions?
- What are your thoughts on dietitians providing a referral to seeing a dentist?
- Do you think dietitians are trained/competent to provide oral health advice?
- How much would you trust this information provided by your dietitian? Why/why not?
- What is your oral health routine
- What are your preventative practices for managing your oral health
- How confident do you feel in dealing with any oral health concerns? Where would you go to seek help? What are the barriers for you in seeking advice or help about oral health concerns?

**Practices/Behaviours**

- When was the last time you visited the dentist?
- What has previously prompted you to visit the dentist? (if the visit was recent)
- How often do you regularly seek dental services for treatment or advice?
- What are the barriers to you accessing oral health care/advice?
- What (if any) are the barriers to you addressing any oral health concerns with health care professionals?
- Has covid impacted your ability to seek health advice or support?
- Are you aware of public dental services and their free services?
- What are the issues with accessing private dentists?

**Resources and oral health promotion**

- What information have they provided you?
- What electronic or written resources have you received and were they useful?
- Have you ever received any oral health promotion material? What information did this provide? Was it useful?
- How would you improve the resources you have received?
- Is there any specific oral health information that you believe would be most useful for someone with an eating disorder?
- How should this information (for the resource) be delivered? E.g electronic resource, written brochure, verbally etc.

Appendix B:

# Researcher positioning statements

**Tiffany Patterson Norrie:** I am a clinical dietitian and PhD candidate who has spent most of my clinical career working in paediatric dietetics. I have ten years of experience working in oral health related research. I have had no previous clinical experience working with individuals with an ED and therefore, do not have any preconceived notions or clinical lens that may have influenced my interaction with participants. It was through my research experience that I made the clinical link between ED and OH, which was further clarified after investigating related literature in more detail.

**Lucie Ramjan:** I am a Registered Nurse and Professor of Nursing. My research expertise is qualitative and has centred on learning more about the personal experiences of treatment and recovery for people with eating disorders. My research supports finding adjuncts to treatment that empower and inspire hope for recovery.

**Mariana S. Sousa:** I am a physician and researcher with clinical and research interests in cancer-associated symptoms, including treatment-related harms and the impact of health on quality of life. My research focuses on better understanding cancer cachexia/anorexia and identifying advances to support patients' nutritional and functional state across the cancer care continuum. It also aims to improve competency of the health workforce to improve outcomes for patients and their families and caregivers.

**Ajesh George:** I am a dentist and Professor of Interprofessional Oral Health. My research expertise is in co-designing and implementing integrated models of oral health care. My research focusses on identifying the oral health needs of at-risk populations and formulating strategies that are acceptable and feasible to implement by non-dental health professionals.
